# Renal Pseudoaneurysm with Associated Arteriovenous Fistula as a Cause of Delayed Bleeding after Percutaneous Nephrolithotomy: A Case Report and Current Literature Review

**DOI:** 10.1155/2023/5103854

**Published:** 2023-07-25

**Authors:** Brecht Devos, Hendrik Vandeursen, Olivier d'Archambeau, Eric Vergauwe

**Affiliations:** ^1^Department of Urology, GZA Hospitals Antwerp, Wilrijk, Belgium; ^2^Department of Interventional Radiology, GZA Hospitals Antwerp, Wilrijk, Belgium

## Abstract

**Background:**

Pseudoaneurysm (PA) with associated arteriovenous fistula (AVF) is a rare delayed bleeding complication, occurring in less than 1% of patients after percutaneous nephrolithotomy (PNL). *Case presentation*. A 54-year-old man underwent PNL on February 28, 2023, for a large renal calculus in the right kidney lower pole, with postoperative delayed bleeding: macroscopic hematuria and bladder clot retention after 3 weeks. An iatrogenic PA and AVF were diagnosed after the failure of conservative measures. The patient was successfully treated with superselective angioembolization (SAE) under local anesthesia.

**Conclusion:**

Late hemorrhagic complications after PNL can be severe. Rapid identification of a renal PA and AVF with SAE has a high success rate and low complication rate, avoiding prolonged hospitalization time and major renal surgery for this patient.

## 1. Introduction

Percutaneous nephrolithotomy (PNL) remains the standard procedure for large renal calculi. Standard access tracts are 24-30 Fr. Smaller access tracts between 12 and 20 Fr, mini PNL, are now increasingly utilized in the adult population and are associated with a lower need for blood transfusion [1]. Delayed bleeding with vascular complications, such as pseudoaneurysm (PA) and arteriovenous fistula (AVF), occurs in less than 1% after PNL [2]. PA is a hematoma surrounded by the adventitia of the artery that occurs because of high-pressure arterial leakage. The blood within the hematoma relates to the artery through an aperture, and this PA has a high risk of rupture. AVF is an abnormal communication between an artery and vein without involving a capillary bed and can cause macroscopic hematuria. The concomitance of PA and AVF is not yet clearly understood. This case report emphasizes the importance of early recognition of PA and AVF after PNL and highlights the physician's dilemma about whether to choose a conservative approach or go for early superselective angioembolization (SAE) in the delayed bleeding setting.

## 2. Case Presentation

A 54-year-old man underwent PNL on February 28, 2023, for a large renal calculus in the right lower pole (largest diameter 2.6 cm, volume 1859 mm^3^) on a noncontrast abdominopelvic CT scan. The stone was located in the right lower pole, and surgery was performed in prone position under general anesthesia. He had coexisting type 2 diabetes mellitus and arterial hypertension, all under control with medications. The collecting system was visualized with contrast through a ureteral catheter, and a posterior lower pole calyx puncture was performed with subsequent Amplatz tract dilatation to 24 Fr over a guidewire. Because the renal calculus was not visualised with the first puncture, a second puncture of an anterior lower pole calyx (24 Fr tract) was performed, and the stone was visualized and fragmented with the ultrasonic Calcuson® lithotriptor. After an uncomplicated procedure, a 22 Fr nephrostomy tube was placed without JJ stent insertion. The operating time lasted 173 minutes. The patient recovered well and was discharged on the 3rd day.

**Figure 1:**
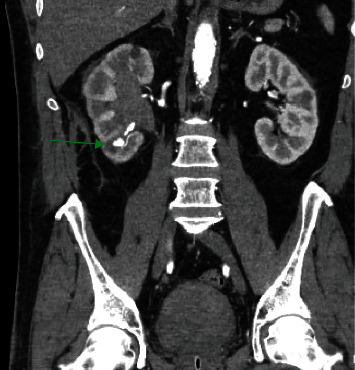
Contrast-enhanced abdominopelvic CT scan showing the arterial phase with contrast stain (green arrow) in the kidney right lower pole, hydronephrosis with hyperdense material in the pyelum, and bladder clot retention.

21 days later, he was transferred from another hospital with macroscopic hematuria and clot retention. Contrast computed tomography angiography (CTA) in the other hospital showed an active arterial bleeding in the kidney lower pole with a contrast stain up to 10.4 mm, hydronephrosis, and hyperdense material in the pyelum, ureter, and bladder (Figure 1). No retroperitoneal hematoma was seen. Upon arriving at our hospital, he was hemodynamically stable; laboratory investigation showed a hemoglobin of 11.2 g/dl and slight acute renal failure.

We repeated the CTA, and after consulting the radiologist, no active bleeding was seen anymore. JJ stent insertion and cystoscopy with clot removal were performed, followed by continuous bladder irrigation. During the hospitalization stay, he remained hemodynamically stable without the need of transfusion. Despite the continuous irrigation, he kept on evacuating blood clots through the transurethral catheter. After a new multidisciplinary consultation meeting between the urologist and radiologist on the 7th day, a new CTA was performed, visualizing a contrast stain of 15 mm in the kidney lower pole with a clear decrease in density in the portovenous phase, suggestive of PA.

**Figure 2:**
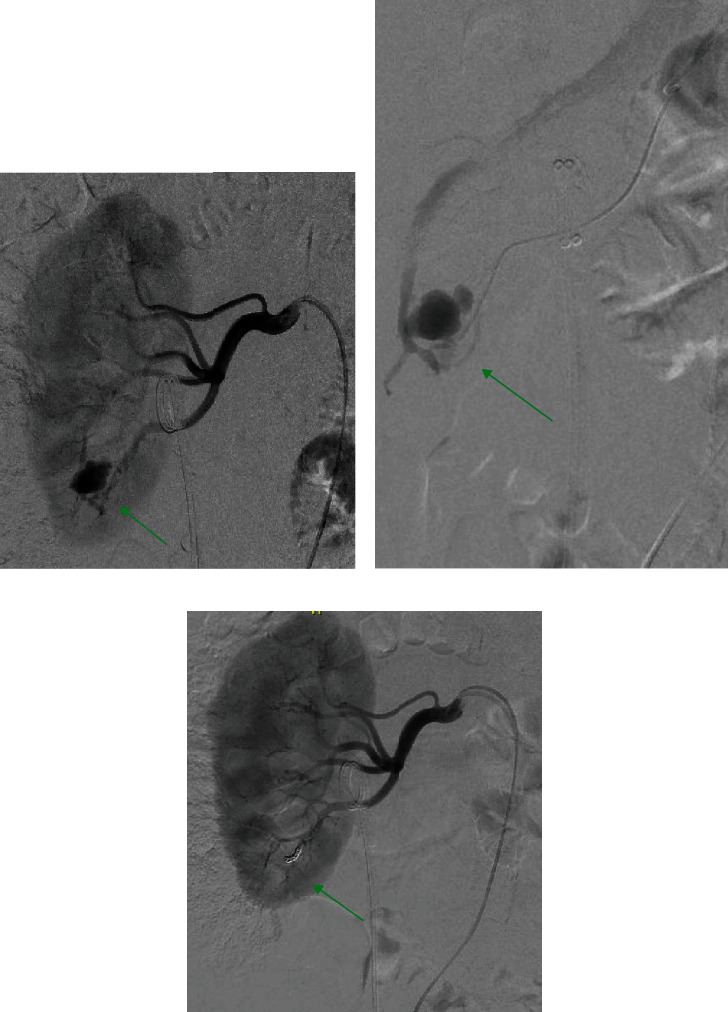
Angiography showing (a) peak opacification of the PA (green arrow) before embolization, (b) superselective catheterization at the base of the PA filled with contrast (green arrow) and the associated AVF with early venous return, and (c) successful obliteration of the feeder segmental artery with 4 Hilal Embolization MicroCoils™ (green arrow) with no residual filling of the PA on control angiography. PA = pseudoaneurysm; AVF = arteriovenous fistula.

A right transfemoral angiography was performed, confirming the PA (Figure 2). The afferent artery was selectively catheterized with a 2.7 Fr Progreat® catheter with visualization of an early venous return, suggestive of PA with associated AVF. The neck of the PA was embolized with 4 Hilal Embolization MicroCoils™ (2 mm diameter, 2 cm length) with good mechanical occlusion and no residual filling of the PA on control angiography. The patient recovered quickly and was discharged on the postoperative 9th day.

## 3. Discussion

Most delayed bleeding after PNL subsides with conservative management, but 1% of cases require intervention [3, 4]. PA and AVF are the main causes of delayed bleeding. This patient had several pre- and intraoperative risk factors for developing hemorrhagic complications: diabetes, arterial hypertension, large stone size, multiple access tracts, and long operative duration [5]. Superselective angioembolization (SAE) is a safe and effective method to control post-PNL arterial bleeding [6, 7]. A skilled interventional radiologist can achieve successful control of bleeding with a variety of agents available. Our treatment preferred materials were microcoils that are more controllable in small vessels and allow a SAE to be as distally as possible, to have minimal parenchymal loss. Theoretically, the concomitance of PA and AVF can increase the technical difficulty of SAE due to the risk of distal dislodgement of embolization material such as coils and particles. However, Barbiero et al. showed in a retrospective multicenter study of 30 cases that there are no significant differences in safety or effectiveness of embolization between PA with or without AVF [8]. A risk factor for potential failure of SAE in this case were the multiple (*n* = 2) 24 Fr access tracts [9].

Shadpour et al. proposed the post-PNL vascular embolization selection (POPVESL) score in a retrospective high-volume single-center study with criteria to predict response to conservative therapy for delayed bleeding from post-PNL intrarenal vascular lesions (Table 1) [2]. When the score is below 11, it correctly predicts the success of conservative management with 81.6% sensitivity and 100% specificity. Conversely, a score above 16 was 100% specific but 52% sensitive for the inevitability of embolization. In our case, the POPVESL score was 8. According to this score, for a patient with stable hemodynamics, it was reasonable to maintain an initial conservative approach.

In a recent retrospective study on a large group of 243 patients with renal artery embolization after PNL, the mean time from PNL to SAE was 6.4 days [10]. Remarkable in our patient was the long time interval (28 days) between PNL and SAE. In a subgroup analysis by Alabat Roca et al. concerning late hemorrhagic complications, the mean time from PNL to SAE in this subgroup was 10.5 days [5]. Although delayed bleeding after PNL is suggestive of a less intense nature, this case illustrates that the sudden onset of, for example, gross hematuria with clots at a certain time point is of more importance than the time interval with subsequent imaging (CTA and angiography) to diagnose intrarenal vascular lesions such as PA and AVF to decide to go for early SAE.

## 4. Conclusion

Late hemorrhagic complications after PNL can be severe. Rapid identification of a renal pseudoaneurysm and arteriovenous fistula with superselective angioembolization has a high success rate and low complication rate, avoiding prolonged hospitalization time and major renal surgery for this patient.

## Figures and Tables

**Table 1:** POPVESL scoring system for calculating the likelihood of requiring vascular intervention for patients with delayed post-PNL bleeding.

P	O	P	V	E
Pseudoaneurysm	Open surgery on the same kidney	Postsurgery interval(≥8.5 days)	Vascular lesion diameter (≥7.5 mm)	Extra units of blood beyond initial stabilization
3 points	5 points	2 points	3 points	2 points per unit

Adapted from Shadpour et al. [2].

## Data Availability

Data sharing is not applicable to this article as no datasets were generated or analyzed during the current study.

## Abbreviations

PNL: Percutaneous nephrolithotomy
PA: Pseudoaneurysm
AVF: Arteriovenous fistula
CTA: Computed tomography angiography
SAE: Superselective angioembolization
POPVESL: Post-PNL vascular embolization selection.
